# Limitations of selective deltamethrin application for triatomine control in central coastal Ecuador

**DOI:** 10.1186/1756-3305-4-20

**Published:** 2011-02-18

**Authors:** Mario J Grijalva, Anita G Villacís, Sofía Ocaña-Mayorga, César A Yumiseva, Esteban G Baus

**Affiliations:** 1 Tropical Disease Institute, Biomedical Sciences Department, College of Osteopathic Medicine, Ohio University. Athens, Ohio, USA; 2 Center for Infectious Disease Research, School of Biological Sciences, Catholic University of Ecuador, Quito, Ecuador

## Abstract

**Background:**

This year-long study evaluated the effectiveness of a strategy involving selective deltamethrin spraying and community education for control of Chagas disease vectors in domestic units located in rural communities of coastal Ecuador.

**Results:**

Surveys for triatomines revealed peridomestic infestation with *Rhodnius ecuadoriensis *and *Panstrongylus howardi*, with infestation indices remaining high during the study (13%, 17%, and 10%, at initial, 6-month, and 12-month visits, respectively), which indicates a limitation of this strategy for triatomine population control. Infestation was found 6 and 12 months after spraying with deltamethrin. In addition, a large number of previously vector-free domestic units also were found infested at the 6- and 12-month surveys, which indicates new infestations by sylvatic triatomines. The predominance of young nymphs and adults suggests new infestation events, likely from sylvatic foci. In addition, infection with *Trypanosoma cruzi *was found in 65%, 21% and 29% at initial, 6-month and 12-month visits, respectively. All parasites isolated (n = 20) were identified as TcI.

**Conclusion:**

New vector control strategies need to be devised and evaluated for reduction of *T. cruzi *transmission in this region.

## Background

Chagas disease in Ecuador affects approximately 230,000 people, and 6.2 million people are at risk of infection by *Trypanosoma cruzi*, its causative agent [1]. In the absence of satisfactory therapy or vaccines against Chagas disease, control of the disease relies primarily on interrupting transmission by eliminating domestic populations of triatomines. Triatomines *Rhodnius ecuadoriensis *and *Panstrongylus howardi *are important vectors of Chagas disease in the Manabí province, located on the central coast of Ecuador, and there is evidence of host (vectors and reservoirs) infected with *T. cruzi*, circulating in the area [2-6].

*R. ecuadoriensis *is widely distributed in Ecuador's central and southern coast [7], its southern Andean region, and in northern Perú [3]. In the coastal region, *R. ecuadoriensis *is usually found in association with *Phytelephas aequatorialis*, an endemic palm species, [5,8] and in nests of squirrel (*Sciurus stramineus*) and bird (*Campylorhynchus fasciatus*) [6]. In this region, there are frequent invasions and colonization by this species in the domestic and peridomestic habitats. However, *R. ecuadoriensis *also is frequently found in domestic and peridomestic habitats in El Oro province, where palm trees are less abundant, and in Loja province and northern Perú, where palm trees are completely absent [2,9,10]. Recent morphological studies have found no differences between sylvatic and domestic/peridomestic populations of *R. ecuadoriensis *in the central coastal region [11], which suggests a constant flow of individuals between these habitats.

*P. howardi *is considered an endemic species of Ecuador, although its distribution seems to be limited to the coastal region [2,12]. The high adaptability of *P. howardi *to different microhabitats within the peridomicile, frequent reports of intradomiciliary invasion and high infection with *T. cruzi *indicate that this species is an important secondary vector [6].

Elements used in housing construction in the Manabí province can influence the likelihood of triatomine infestation. For example, sometimes, roofs are constructed with leaves of *P. aequatorialis*, a common environment for triatomines to colonize. However, in recent years, the use of zinc plates as roofing material has become common [13]. This material does not provide an environment in which triatomines can become established. Also, houses are elevated by wooden stilts, and walls and floors are made of guadúa cane (*Guadua angustifolia*). Although bare cane features large gaps that allow passage by insects and does not offer hiding places for the bugs, when cane is covered with paper or other materials, it may become a suitable hiding place for triatomines.

The peridomestic area in the Manabí province also offers triatomines plenty of potential microhabitats and opportunities for colonization by synanthropic animals. For example, the peridomicile is characterized by an abundance of *P. aequatorialis*, which is cultivated for its nuts used in the manufacture of handcrafts and buttons that constitute an important source of income for the rural population. Other palms, such as coconut and African palms (*Elaeis guineensis*) are also commonly found near houses [14]. In addition, chicken coops, guinea pig pens, agricultural and household waste, the thorny "piñuela" plant *Aechmea magdalenae *and other plants used as natural fences, piles of construction materials, and accumulations of palm fronds provide good refuges for triatomines. Finally, the definition of peridomestic habitat becomes blurred because of the frequent close proximity of brush and jungle to the house.

Insecticides are one of the main tools used for control of vector-borne diseases [15]. Synthetic pyrethroids such as deltamethrin, cypermethrin, cyfluthrin, and lambdacyhalothrin are commonly used for Chagas disease control [16,17]. The aim of the Chagas Control Programs is to eliminate domestic colonies of triatomines. Indeed, application of deltamethrin only to houses found infested by triatomines (known as "selective application" as opposed to "community-wide application") for the control of Chagas disease in the southern Andean region of Ecuador has shown a notable reduction of household infestation (from 12.5% to 3.8%), especially by *R. ecuadoriensis *and *Triatoma carrioni *(Grijalva MJ, unpublished data), as well as a reduction in operational costs.

Despite the success of insecticide-based strategies to control domestic triatomine infestations, the effectiveness of these interventions is hampered in areas where sylvatic triatomine populations occur and readily invade treated houses when residual insecticide activity declines [18,19]. Here we report findings of a study designed to evaluate the effectiveness of selective deltamethrin spraying and community education in rural communities of the Ecuadorian central coast.

## Results

### Domiciliary units

A total of 230 DUs was examined at least once in the four surveyed communities in the Manabí province, Ecuador. Of these, 187 were examined during the first visit. However, it was not possible to re-examine all 187 due to the following reasons: (1) the head of household declined to continue participation in the study, (2) the head of the household was absent at the time of follow-up visits, or (3) the DUs became uninhabited between study visits. The coverage of entomological examination was 81.3%, 63.3% and 84.9% at the initial visit, 6 months, and 12 months, respectively. Only 109 DUs were examined all three times.

All communities had similar house construction, peridomicile characteristics and economic activities. Most houses had a closed roof (84%), bare cane walls (70%), and wood board floor (66%) (Table 1). Houses tended to be small (≤2 bedrooms), and although most had electricity, 11% of houses did not have any form of latrine or public sewer system. Indoor storage of firewood and agricultural products was infrequent, and 55% of houses reported insecticide application by the Malaria Control Program within the 12 months prior to our initial visit. Detailed information about prior insecticide application was not available. Peridomestic characteristics, including livestock farmed, are summarized in Table 2. In this habitat, piles of firewood, presence of piñuelas *(Aechmea magdalenae)*, fruit trees, brush, and bushes near the dwelling were the most representative characteristics. Chickens were present in 93% of DUs.

**Table 1:** Construction materials and other characteristics of 230 houses located in four rural communities of the Manabí province, Ecuador

House characteristics	(%)
Roof	
Cement/Asbestos/Zinc	84
Tile	2
Other, including palm	14
Type of Walls	
Cement/brick	21
No paneled cane	70
Paneled cane*	9
Other	2
Type of Floor	
Cement/Tile/Wood parket	16
Wood boards	66
Cane, other	30
Dirt	5
Size	
Bedrooms < = 2	63
Services	
Electricity	93
No latrine	11
Piped water	5
Cooking fuel	
Natural gas	86
Firewood/Coal	66
Intradomicile storage	
Firewood	6
Agricultural products	4

^a^Cane walls are sometimes covered with paper or other materials

**Table 2:** Peridomestic materials, livestock, and vegetation of 230 domestic units located in four rural communities of the Manabí province, Ecuador

Item	Frequency (%)	Average per house
*Materials accumulated*		*Distance from house in meters. Mean (SD)*
Firewood	55	7 (6)
Wood for construction	31	6 (5)
Rock/brick piles	21	6 (6)
Household trash	16	10 (7)
Tagua palm frond piles	14	8 (6)
Coconut palm frond piles	6	8 (3)
Agricultural refuse	8	12 (9)
Agricultural products	24	8 (5)
**Vegetation**		
Brush or scrub	61	9 (6)
Piñuelas (*A. magdalenae*)	51	11 (6)
Bushes (arbustos)	71	9 (6)
Fruit trees	63	7 (5)
Coconut Palms	33	10 (7)
Tagua palms	17	13 (8)
***Domestic animals***		***Number of animals per house***
Chikens other birds	93	26 (22)
Chicken pen	51	NA
Dogs	74	2 (2)
Guinea pigs	9	5 (4)
Guinea pig pen	8	NA
Pigs	51	3 (2)
Sheep or goats	3	6 (3)
Sheep/goat corral	10	NA

NA: No applicable

### Baseline triatomine infestation

**Figure 1:**
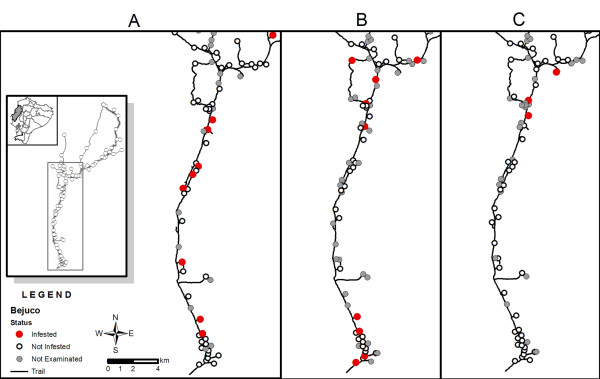
**Map of a section of Bejuco community, indicating the distribution of triatomine infestation of domestic units (DUs) at the A) initial, B) 6-month and C) 12-month visits**. Insert panels represents the location of the study site and the Manabí province within Ecuador, a map of the location of all DUs of Bejuco community indicating the section of the community shown in detail. Open, closed red and greyed circles represent non-infested, infested and non examined DUs, respectively.

Triatomines were found in 13% of the 187 DUs examined. Of the 109 DUs examined three times, 16 (15%) were infested with triatomines (Table 3). A total of 195 live individuals of *R. ecuadoriensis *and 24 of *P. howardi *was collected in peridomestic habitats; neither species was found inside the house. In addition, a total of 22 dead specimens (14 *R. ecuadoriensis *and 8 *P. howardi*) was collected. Most of the live *R. ecuadoriensis *were associated with chicken nests, guinea pig pens, wood piles, and rat nests in piñuela plants. Most *P. howardi *were found in wood, rock, and bricks piles and in rat nests within piñuelas. Of the 16 DUs from which triatomines were recovered, three contained both species of triatomine but in different microhabitats. The overall density of triatomines was 2.0 ± 6.3 bugs per searched DU; crowding was 13.7 ± 10.6 bugs per infested DU; and colonization was 94% (Table 3). As an example, distribution of infested DUs in a representative zone of El Bejuco community can be seen in Figure 1a. The triatomine infestation at the initial visit was higher in DUs that self reported prior insecticide application by the Malaria Control Service than in those that did not report prior treatment (10 and 6 DUs, respectively). However, no statistical association was found between pretreated and non-pretreated DUs and infestation (p > 0.05). None of the 109 DUs examined tree times was found to be infested in all the visits.

**Table 3:** Triatomine infestation found over three visits in four rural communities in Portoviejo county, the Manabí province, Ecuador*

	***Rhodnius ecuadoriensis***			***Panstrongylus howardi***			**Both triatomines**		
	
	**Initial visit**	**6 month**	**12 month**	**Initial visit**	**6 month**	**12 month**	**Initial visit**	**6 month**	**12 month**
			
Houses infested^¥^	**14**	**13**	**11**	**5**	**6**	**0**	**16**	**16**	**11**
Infestation index (%)	13	12	10	5	5	0	15	15	10
Density index	1.8 ± 6.0	1.0 ± 0.3	1.5 ± 7.1	0.2 ± 2.8	0.2 ± 1.0	-	2.0 ± 6.3	1.21 ± 4.3	1.5 ± 7.1
	(0--38)	(0--29)	(0--58)	(0--17)	(0--7)	-	(0--38)	(0--29)	(0--58)
Crowding index	13.9 ± 10.9	8.5 ± 9.0	14.7 ± 18	4.8 ± 5.8	3.5 ± 2.8	-	13.7 ± 10.6	8.3 ± 31.1	14.7 ± 18
	(1--38)	(1--29)	(1--58)	(0--15)	(0--7)	-	(1--38)	(1--29)	(1--58)
Colonization index (%)	93	62	91	100	50	0	94	63	91

* Includes only 109 domestic units that were examined in each of the 3 visits. In each visit with permission of the head of the household manual triatomine searches were conducted in the house and in the peridomicile using the one man/hour method.

^¥ ^Infestation is defined as finding at least one live triatomine. If found infested at any visit houses and peridomiciliary areas were sprayed with deltamethrin at 25 mg a.i./m^2^. Entomological indices: Infestation index (100 × number of DUs infested/number of DUs searched), density (number of triatomines captured/number of DUs searched) ± standard desviation (SD) and range, crowding (number of triatomines captured/number of DUs infested) ± SD and range, and colonization index (100 × number of DUs with nymphs/number of DUs infested).

#### Population structure

**Figure 2:**
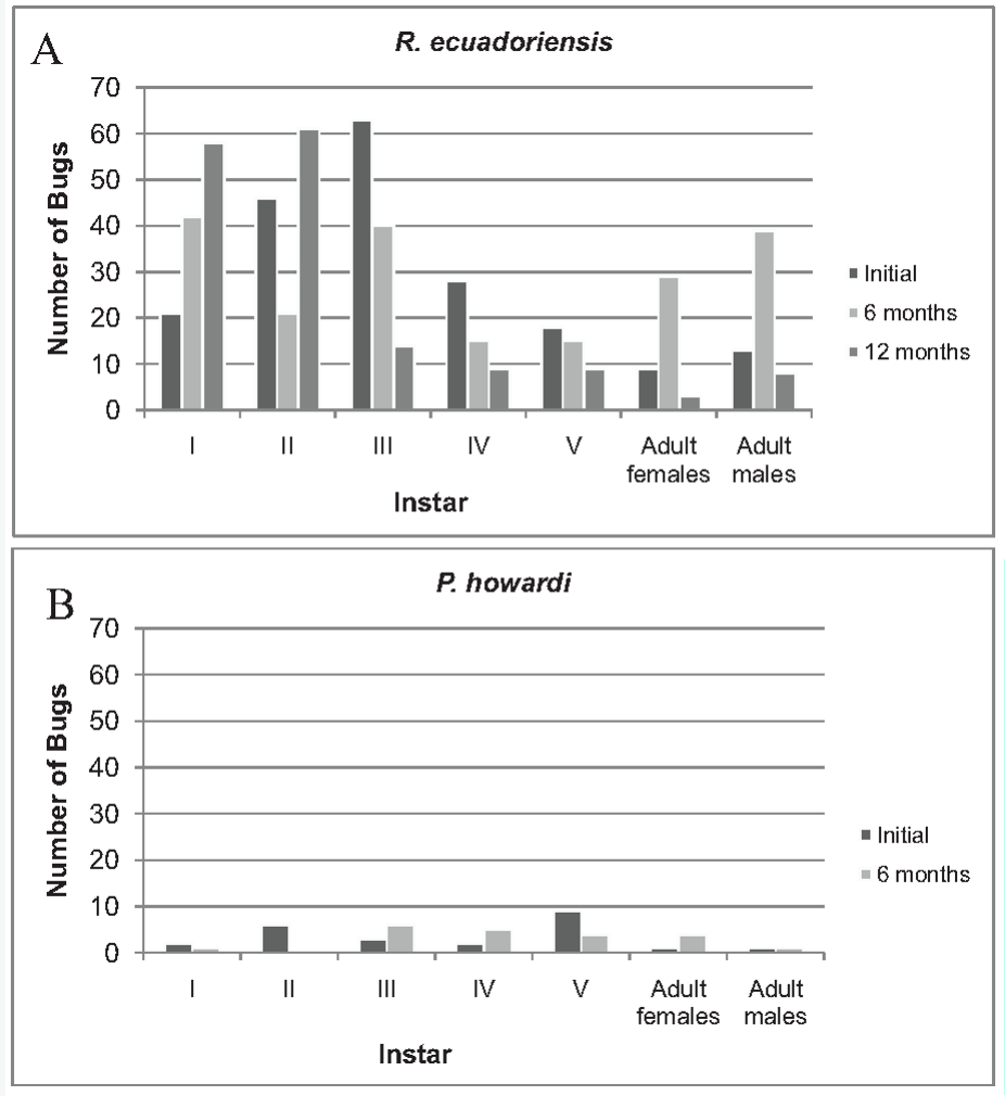
**Population structure of A) *R. ecuadoriensis *and B) *P. howardi *found infesting domiciliary units in four rural communities of the Manabí province, Ecuador, in an initial survey or follow-up surveys 6 and 12 months later**.

In the initial visit all instars of *R. ecuadoriensis *were found; however, instars I--IV were more abundant than instars V and adults (females and males) (Figure 2a). The species *P. howardi *was less abundant, yet all instars were found (Figure 2b).

### Triatomine infestation at 6 months

Triatomines were found in 17% of a total of 149 DUs examined during this visit (14% with *R. ecuadoriensis *and 5% with *P. howardi*). Of the 129 DUs examined at the initial and 6-month visits, 22 (17%) contained triatomines. Infestation indices for each community can be seen in Table 3. As in the initial visit, triatomines of both species were found mainly in the peridomicile (240 *R. ecuadoriensis *and 23 *P. howardi*), and just two adult male individuals of *R. ecuadoriensis *were collected inside houses. *R. ecuadoriensis *was found in association with wood, chicken and rat nests and accumulations of tagua palm fronds. Inside the house, the bugs were found in the bedroom. *P. howardi *was associated mainly with piles of bricks, wood, garbage, and rat nests. Triatomine density and crowding were 1.21 ± 4.3 and 8.3 ± 31.1 respectively, but a reduction in the colonization index was observed (94% to 63%, Table 3). Dead bugs of both species were collected in the house (4 specimens) and peridomicile (16 specimens).

#### Infestation

**Figure 3:**
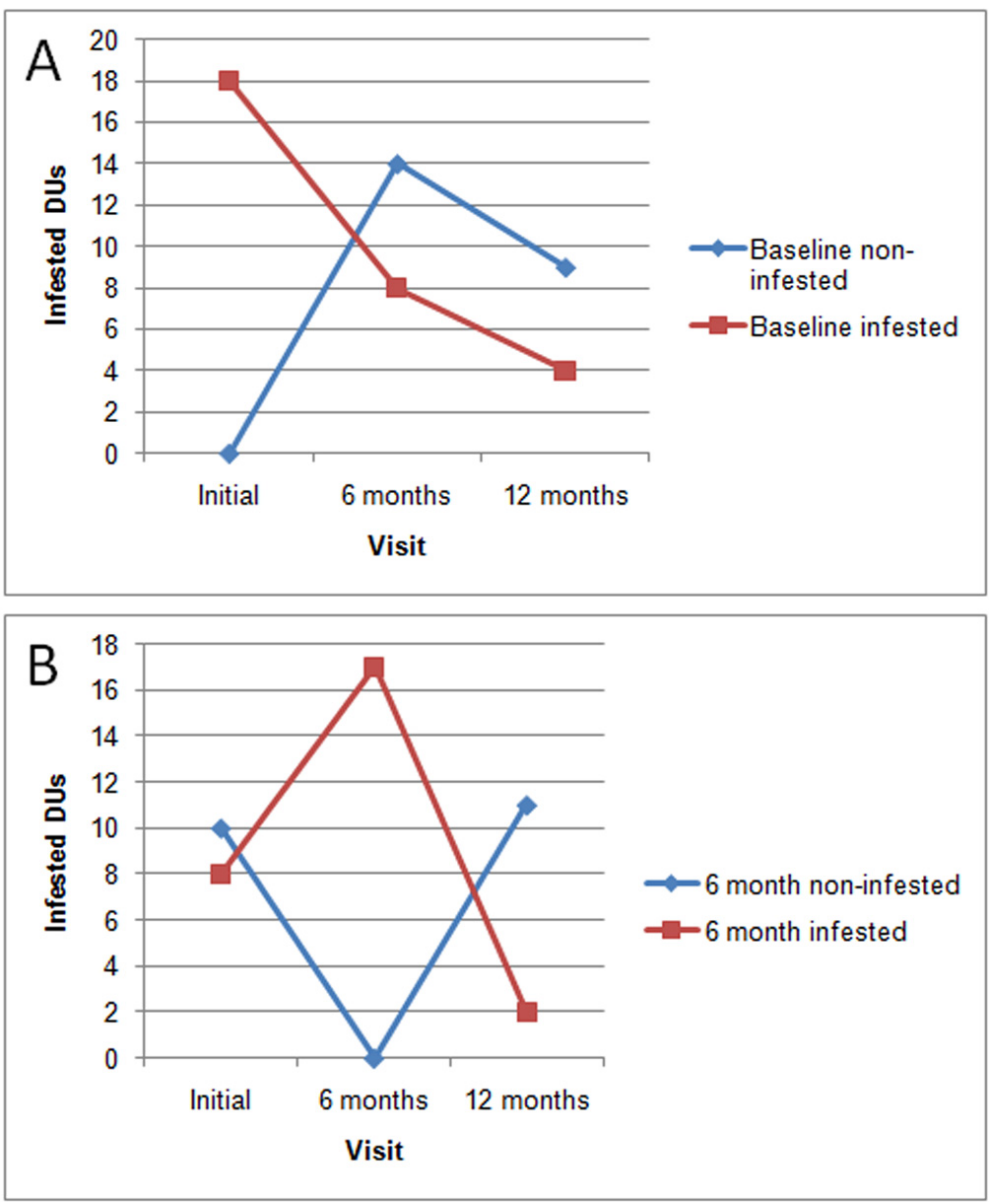
**Domestic Units infested against time**. Lines follow separately over the duration of the project those groups of domestic units that were found infested and not-infested at the A) baseline and B) six month visit. Recurrent infestation is represented by orange squares and new infestations are represented by blue diamonds.

Of the 22 DUs infested at 6 months, eight of them (five with *R. ecuadoriensis*, two with *P. howardi*, and one with both species) met the definition of reinfestation. The other 14 infested DUs had not been previously infested (Figure 3a). Among the reinfested DUs, most specimens of *R. ecuadoriensis *(102 of 132 individuals) were collected in chicken nests at one DU. In the remaining reinfested DUs, triatomines were found in chicken nests and rat nests within firewood piles. Only three DUs were reinfested with *P. howardi*, and with a low number of bugs. Variation in distribution of infested DUs in El Bejuco can be seen in Figure 1b.

#### Population structure

For *R. ecuadoriensis*, more instar I and adult (male and female) specimens were found at this visit than at the initial visit (Figure 2a). For *P. howardi*, a predominance of instars III, IV, and V and adult females was found (Figure 2b).

### Triatomine infestation at 12 months

A total of 195 DUs was examined, of which 9% were found to be infested with *R. ecuadoriensis *and 0.5% with *P. howardi*. Among the 109 DUs visited three times, only *R. ecuadoriensis *was found, with an infestation index of 10% (Table 3). No triatomines were found in houses, whereas in the peridomicile, *R. ecuadoriensis *was associated with chicken nests, guinea pig pens, wood piles, and rat nests. The triatomine density and crowding were similar to previous visits, but the colonization indices increased to levels similar to the one found at the initial visit (Table 3). *R. ecuadoriensis *eggs (n = 36) and dead individuals were collected in houses (n = 4), and in the peridomicile (n = 24). Residents of the houses gave the research team eight male specimens of *P. howardi *that had been attracted by light at night; however, these were excluded from the analysis.

#### Infestation

Of the 13 infested DUs, four had been infested after being sprayed with deltamethrin at the initial visit (Figure 3a) and two had been infested after spraying at the 6-month visit (Figure 3b). Interestingly, 11 DUs found to be non-infested at 6 months were infested at 12 months (Figure 3b). All DUs were infested only with *R. ecuadoriensis*, which was found mainly in firewood piles, mice nests, and chicken nests. The remaining five DUs found infested at the 12-month visit had not been infested before (Figure 3a and 3b). Figure 1c shows distribution of infested DUs in a representative area of El Bejuco at the 12-month visit.

#### Population structure

A notable increase in the proportion of *R. ecuadoriensis *instar I and II was evident (Figure 2a). Instar I was predominant in reinfested DUs that were infested at the initial visit and 6 -month visit (10 out of 20 triatomines found). Similarly, in reinfested DUs that were infested at 6 months only, 23 out of the 32 found were instar I.

### Natural infection with trypanosomes

Of the 78 triatomines examined, 51% presented infection with *T. cruzi *and 9% infection with *T. rangeli*. The data by vector species indicates single infection with *T. cruzi *in 41% of *R. ecuadoriensis *(n = 64 bugs examined) and 79% of *P. howardi *(n = 14). In addition, single infection with *T. rangeli *was detected only in *R. ecuadoriensis *(6%), however, mixed *T. cruzi*/*T. rangeli *infections were found in 3% and 7% of *R. ecuadoriensis *and *P. howardi*, respectively. The data by visit indicates higher infection rates with *T. cruzi *in the initial visit for both triatomine species. However, due to high mortality in the field no *P. howardi *were analyzed at 6 and 12 months. *T. cruzi *infection over time can be seen in Table 4. A total of 20 samples of *T. cruzi *was characterized as belonging to the TcI lineage.

**Table 4:** Percentage of *T. cruzi *and *T. rangeli *infection by visit in triatomines collected in four rural communities of the Manabí province, Ecuador

	*Rhodnius ecuadoriensis*				*Panstrongylus howardi*				Both triatomines			Total
	
	Initial visit	6 month	12 month	Total	Initial visit	6 month	12 month	Total	Initial visit	6 month	12 month	-
	
*n*	38	19	7	64	14	-	-	14	52	19	7	78
*T. cruzi*	52.6	21.1	28.6	40.6	78.6	-	-	78.6	59.6	21.1	28.6	47.4

*T. rangeli*	7.9	5.3	-	6.3	-	-	-	-	5.8	5.3	-	5.1

Mixed infection	5.3	-	-	3.1	7.1	-	-	7.1	5.8	-	-	3.8

### Infestation and reinfestation

A statistic contrast to test for overall differences in the infestation indices among visits was found not significant (p = 0.09) based on the ALR analysis. PORs from this analysis are shown in Table 5. Only the POR relating the baseline and 6-month evaluations from the same DUs were significant. This indicates that DUs infested at baseline are more likely to be infested at 6 months (p = 0.002). This association was not apparent between the other evaluations. However, an examination of the response patterns indicated that 8 of 18 DUs that were infested at baseline were also infested at 6 months. In addition, 14 of the 111 DUs that were not infested at baseline were infested at 6 months. A similar situation was found between visits at 6 and 12 months: 2 of the 17 houses infested at 6 months remained infested at 12 months, and 11 of 103 DUs not infested at visit 6 months were infested at 12 months. No statistically significant bivariate associations were found between house construction or peridomicile characteristics and infestation with either of the triatomine species found (P > 0.05).

**Table 5:** Pair wise odds ratios for within DU infestation evaluations

Evaluations	Pairwise Odd Ratio	95% CI	p-value
Baseline and 6 months	5.5	1.8, 16.6	0.002
Baseline and 12 months	3.0	0.8, 11.0	0.100
6 and 12 months	1.3	0.2, 7.0	0.768

## Discussion

The results obtained by surveying for triatomines immediately before and 6 and 12 months after selective deltamethrin spraying combined with educational activities have shown that this intervention is not an effective approach for controlling peridomestic triatomine populations in the coastal province of Manabí, Ecuador. We conclude that the high rate of reinvation from nearby sylvatic habitats combined with the re-emergence of triatomine residual population are responsible for maintaining high triatomine infestation in the Manabí province. Therefore, alternative interventions need to be developed to improve insecticide application effectiveness and prevent triatomine infestation in regions where well established sylvatic triatomine populations are present.

Entomological data at baseline showed a thriving peridomestic triatomine population in the study area, containing all developmental instars. Infestation was characterized by a high colonization index of peridomestic microhabitats that are common in most rural DUs in the region [6]. Infestation and entomological indices at 6 and 12 months after the intervention were similar to baseline, indicating little or no effect of the activities in controlling triatomines or, therefore, the risk of transmission. This was emphasized by the observation that at both time points triatomine infestation included infestation of previously treated DUs as well as new infestations and that high infection rates with *T. cruzi *were determined in all visits, especially of *R. ecuadoriensis*. In addition, infestation at Baseline was not associated with self reported prior application of insecticide by the Malaria Control Program within the previous 12 months. Typically, mosquito control in the area consists of indoor only application Malathion. It is possible that prior application of this insecticide played a role in the low intra-domicile infestation found. However, lack of detailed information about these previous interventions prevented us to conduct detailed analyses.

It is possible that some of the triatomines found at 6 and 12 months, particularly *P. howardi*, were survivors of the initial population infesting those DUs. This is supported by the association shown between triatomine infestation at the initial and 6-month time points. Short lived residual activity or incomplete penetration of the insecticide could account for persistent bug populations [20]. However, our results also show a population structure with a predominance of young nymphs and adults, and infestation of previously not infested DUs, suggesting new infestation events. Considering the documented sylvatic triatomine abundance in this area [6], new infestation could be driven by active colonization and/or passive transport of bugs from the forest into wood piles and mice nests. In addition, it is possible that there is recolonization by sylvatic triatomines of available peridomestic microhabitats in DUs that were previously treated. This could occur soon after the residual effect of the pyretroid wanes [20]. Nevertheless, our data, together with our previous reports that sylvatic and peridomestic populations of *R. ecuadoriensis *appear not to be different morphometrically in the same region, suggest a constant flow of individuals between these populations [11]. This scenario seems to be feasible, and preliminary information from the genetic analysis of *T. cruzi *indicates all isolates belong to the TcI lineage, regardless the habitat and the species of host. Unfortunately, small parasite isolate sample size prevents us to draw conclusions regarding genetic similarity of parasites from triatomines collected in reinfested DUs. A larger study is ongoing to determine *T. cruzi *population dynamics in the Manabí province.

Post-intervention infestation with *P. howardi *was lower than for *R. ecuadoriensis*, perhaps as a consequence of *P. howardi*'s smaller sylvatic population. Indeed, while an abundance of sylvatic *R. ecuadoriensis *has been reported in association with palms, squirrels, mice, and bird nests, only one report exists of sylvatic *P. howardi*, which was found associated with mouse nests in the spiny palm *Aiphanes eggersii *and the epiphyte *Bromelia penguin *[6].

Previous studies have shown that suspension concentrated pyretroid spraying using manual compression sprayers are more effective than emulsificate concentrate using power sprayers [20]. However, our data indicates that the use of WP deltamethrin using manual compression sprayers was not effective in controlling peridomestic triatomines. As previously reported, rain, dust and sunlight quickly reduce the residual effect of the insecticide and its efficacy to prevent reinfestation of peridomestic structures [20]. In addition, treating large piles of bricks, rocks, lumber, and palm fronds using compression sprayers is difficult: the process requires intense physical activity and involves moving the materials so that insecticide can be thoroughly sprayed. As a result, this sort of treatment might not be applied effectively, and the effort to do so could be demoralizing to field personnel. Thus, this type of insecticide application is not practical on a large scale, and the effectiveness of different application methodologies should be evaluated. Some possibilities include the use of ultra-low-volume foggers that could be used to apply insecticide at 40 mg a.i./m^2 ^to large piles of materials covered with heavy plastic. Another alternative is the use of micro-encapsulated formulations of organophosphates applied which has recently shown long-lasting residual effect when used in domestic and peridomestic habitats [21].

Historically, *Triatoma dimidiata *has been considered the main Chagas disease vector in coastal Ecuador [2], but we found no evidence of its presence in the study area. This could be due to effective elimination of this introduced species. It is also possible that in many previous field reports, *P. howardi *could have been misidentified as *T. dimidiata *as the two share remarkably similar dorsal pigmentation. Unfortunately, this possibility cannot be investigated due to the lack of archival specimens.

Transmission of Chagas disease in the central coast of Ecuador is not yet understood, but the environment in which triatomines are found could give us important clues. In the case of *R. ecuadoriensis*, chicken nests are among the first microhabitats to be colonized. Besides the clear implication for Chagas disease transmission, a large number of bugs feeding on roosting chickens might have an impact on the birds' nutritional state and reduce their weight gain and egg production. Therefore, control strategies should include educating residents about the need to monitor nests for bugs, avoid use of banana leaves or other vegetal material for construction of coops, and change nest materials regularly, burning the old material to eliminate bugs and eggs. In addition, people should examine chickens for the presence of bugs under their wings or other body parts before transporting them for commerce. Finally, alternatives should be investigated for safely treating chickens with insecticides to prevent them from becoming infested [22].

The main limitation of this study was the loss of the 78 DUs that were not each sampled three times. Most of the dropouts were due to the absence of the head of the household at the time of the visit. However, an important number of dropouts were due to refusal of entomological search. This is understandable as searches involve time commitment and an invasion of privacy. Interestingly, some of these households and some that had initially refused to participate requested re-instatement or enrolment in the project at 12 months. These requests could be the result of a combination of the community education activities conducted during each visit and word-of-mouth dissemination of the finding of new and recurring triatomine infestation. This study was originally designed to include a risk factor analysis of households; that analysis was not possible due to the relatively small sample size. However, research is ongoing to describe the distribution of domestic and sylvatic triatomine infestation throughout the Manabí province and to understand seasonal variations on triatomine density and migration. Studies are also looking at genetic relationships between vector and trypanosome populations in this region.

## Conclusions

In summary, our investigation provides strong evidence that the use of selective deltamethrin spraying and community education is not enough to control peridomestic triatomine populations in the Manabí province. The high rate of triatomine infestation observed, even in peridomiciles that had been sprayed with insecticide, needs to be considered foremost in designing and implementing Chagas disease control interventions in this area. New insecticide delivery methods need to be evaluated in conjunction with community education and frequent active and passive surveillance of domestic, peridomestic, and sylvatic habitats.

## Methods

### Study area

The Manabí province is located in the central coast of Ecuador and has an average annual rainfall of 563 mm/year [23]. The main economic activity of this area is agriculture, with a predominance of sugar cane, oranges, banana, yucca, corn or maize, and rice. In addition, some palms, such as the tagua palm *(P. aequatorialis)*, coconut palm (*Cocos nucifera *L.), and African palm (*Elaeis guineensis*) are cultivated in this region.

This study was carried out in four rural communities in Portoviejo county: Bejuco (S0.9495°, W80.3314°, 65--400 meters above sea level [masl], n = 106 domestic units [DU]), Maconta Abajo (S01.0808°, W80.4125°, 68--144 masl, n = 53 DU), Jesús María (S01.0203°, W80.2282°, 65--400 masl, n = 54 DU), and San Gregorio (S01.031°, W80.2373°, 74--220 masl, n = 50 DU).

#### Household surveys

All houses in each community were visited by two-person teams of trained field workers from the National Chagas Disease Control Program who conducted the triatomine searches. They were accompanied by students and staff from Catholic University of Ecuador and/or students participating in education abroad programs organized by the Tropical Disease Institute at Ohio University. These students and staff supervised and assisted in the administration of a questionnaire and educational activities. Written informed consent for participation was obtained from the head of each household following a protocol approved by the Ohio University and Catholic University of Ecuador Institutional Review Boards.

The initial visit was conducted in March 2007 to establish the baseline for the study. Second and third visits were conducted 6 and 12 months later. Some houses were found to be closed at the time of visit; some households chose to end participation in the study; and other households were enrolled upon request during the 6- and 12-month visits. During the initial visit, a questionnaire was administered to the head of the household to document construction materials and other characteristics of the domicile and peridomicile. During each visit, an educational talk that included information about Chagas disease, triatomine habits, and prevention of Chagas disease was given to the family members present at each house. The talks were reinforced by educational booklets that were given to each member of the household and to all school children. This message was reinforced by the presentation of educational videos at the schools.

### Triatomine collection in domestic and peridomestic habitats

Each domestic unit (DU), which included house and peridomicile, was searched by a two man team for triatomines using the one man-hour method. After 20 minutes, if no live bugs were found, a dislodgant agent (6% aqueous pyrethrin [ExciteR™, Prentiss Inc., Sandersville, GA]) was hand sprayed into cracks, roof space and other hard to reach areas, and the search was continued for 10 more minutes [17]. The peridomicile was defined as all surrounding structures and potential habitats within 20 m of the house [10].

All live and dead triatomines, eggs, and exuviae that were found were placed in individually labelled plastic flasks. Identification of taxonomy and developmental stage was done at the insectary of the Center for Infectious Disease Research at the School of Biological Sciences in the Catholic University of Quito-Ecuador based on the keys by Lent and Wygodzinsky and Carcavallo *et al. *[24,25].

### Deltamethrin intervention

In DUs found to be infested, permission to spray insecticide was sought from the head of the household, and the house was prepared by removal of all food, kitchen utensils, bedding, clothes, and moveable house ware. The study protocol specified complete indoor and outdoor spraying of all dwellings and peridomestic structures with deltamethrin WP 25 mg a.i./m^2 ^using backpack sprayers (Hudson X-pert) and appropriate personal protection equipment as previously described [17]. Residents were asked not to enter the dwelling for at least one hour. To prevent environmental pollution at the end of each day, any remaining insecticide and the water used for cleaning the sprayers were stored for preparation of the first charge used the next day [17].

### Entomological indices and trypanosome infection

A domicile or peridomicile was considered infested if at least one live triatomine was found. The following entomological indices were calculated for each visit: Infestation index (100 × number of DU infested/number of DU searched), density (number of triatomines captured/number of DU searched), crowding (number of triatomines captured/number of DU infested), and colonization index (100 × number of DU with nymphs/number of DU infested) [15]. Reinfestation was defined as the presence of live triatomines in a DU after it had been found infested at a previous visit and treated with insecticide. The source of the recurring infestation (survivors or true immigrants from sylvatic habitats or outside the studied) area was not determined [17].

Natural trypanosome infection was determined in 78 randomly chosen triatomines by microscopic observation and PCR using S35/S36 primer sets as previously described [26]. The natural infection index (100 × number of triatomines with *Trypanosoma sp*/number of studied triatomines) was calculated for *T. cruzi, T. rangeli*, and mixed infections. The lineage of 20 *T. cruzi *isolates was determined as previously described [27].

### Statistical analyses

Descriptive statistics were calculated for all variables. We modeled the probability of infestation across time points using an alternating logistic regression (ALR) model [28], an alternate form of generalized estimating equations (GEE). ALRs model both the patterns of clustering and the marginal probabilities of infestation. Pair wise odds ratios (POR) were used to model the association between infestation evaluations across time points within DUs. These PORs allowed us to assess whether DUs infested at a given time are more likely to be found to be infested at later assessments. In addition to accounting for the correlation among repeated measurements, ALRs use incomplete data the same way as other methods for the analysis of repeated measures, such as generalized estimating equations (GEE) and models with random effects. Chi-square was used to test the relationship between household characteristics and baseline infestation data for each species. No multivariate analyses were conducted due to the scarcity of statistically significant bivariate associations. ALR analysis was performed using SAS 9.3. All other statistical tests were carried out using SPSS 17.0.

## Competing interests

The authors declare that they have no competing interests.

## Authors' contributions

MJG: conceived of the study, and participated in its design and coordination, performed data analyses and helped to draft the manuscript. AGV: directed entomological collection, triatomine identification, data analyses and drafter the manuscript. SO conducted parasite isolation, molecular characterization and helped to draft the manuscript. EGB participated in the data collection, management and analyses. CAY participated in data collection, operated the geographical information system and data management and produced the maps. All authors read and approved the final manuscript.
